# Molecular phylogeny of bark and ambrosia beetles reveals multiple origins of fungus farming during periods of global warming

**DOI:** 10.1186/1471-2148-12-133

**Published:** 2012-08-01

**Authors:** Bjarte H Jordal, Anthony I Cognato

**Affiliations:** 1 Natural History Museum, University Museum of Bergen, NO-5020, Bergen, Norway; 2 Department of Entomology, Michigan State University, 243 Natural Science Bldg, East Lansing, MI, 48824, USA

**Keywords:** Agriculture, Symbiosis, Molecular phylogeny, Bark and ambrosia beetles, Evolutionary origins

## Abstract

**Background:**

Fungus farming is an unusual life style in insects that has evolved many times in the wood boring weevils named ‘ambrosia beetles’. Multiple occurrences of this behaviour allow for a detailed comparison of the different origins of fungus farming through time, its directionality, and possible ancestral states. We tested these hypotheses with a phylogeny representing the largest data set to date, nearly 4 kb of nucleotides from *COI*, *EF-1α*, *CAD*, *ArgK*, *28S*, and 200 scolytine taxa.

**Results:**

Phylogenetic analyses using Bayesian or parsimony approaches placed the root of Scolytinae close to the tribe Scolytini and *Microborus*, but otherwise indicated low resolution at older nodes. More recent clades were well resolved, including ten origins of fungus farming. There were no subsequent reversals to bark or phloem feeding in the fungus farming clades. The oldest origin of fungus farming was estimated near 50 Ma, long after the origin of Scolytinae (100-120 Ma). Younger origins included the species rich Xyleborini, dated to 21 Ma. Sister group comparisons and test of independence between traits indicated that neither gregarious larval feeding nor regular inbreeding by sibling mating was strongly correlated with the origin of fungus farming.

**Conclusion:**

Origins of fungus farming corresponded mainly with two periods of global warming in the Cenozoic era, which were characterised by broadly distributed tropical forests. Hence, it seems likely that warm climates and expanding tropical angiosperm forests played critical roles in the successful radiation of diverse fungus farming groups. However, further investigation will likely reveal additional biological factors that promote fungus farming.

## Background

Bark and ambrosia beetles in the weevil subfamily Scolytinae are remarkably diverse in their ecological adaptations to a variety of habitats [1]. With more than 6,000 species currently recognised, they comprise about 10 percent of the total weevil diversity, and thus constitute one of the greatest species radiations on earth [2]. Scolytine beetles are generally enormously abundant and dominate forest insect communities associated with recently dead wood and other lignified plant material [3-5]. Their ubiquitous presence in nearly all woody material indicates that these beetles are the most important organisms in the early stages of forest decomposition. In these habitats they excavate diverse tunnel systems reflecting different reproductive modes and variable diets in different plant tissues. A large scale phylogeny of Scolytinae will therefore illuminate many aspects of key evolutionary traits in this group of beetles.

One of the most successful ecological adaptations in bark and ambrosia beetles is their symbiotic relationship with microbes. This relationship provides nutritional enrichment of dead plant material in exchange for transmission between plant resources. These microbes include bacteria, yeast and mycelial fungi. While the exact role of the first two is not known [6,7], the multicellular fungi contribute an important but variable component of a wood boring beetle’s diet [8,9]. In about 2,000 species of Scolytinae, Ophiostomales and Microascales fungi are the sole source of food for both larvae and adults and are actively cultivated by the beetle. This extraordinary agricultural system shares many similarities with fungus farming in ants and termites [10-12]. These are the ambrosia beetles and they include common pest species such as the striped ambrosia beetle *Trypodendron lineatum* and the red bay ambrosia beetle *Xyleborus glabratus*. In addition to scolytine ambrosia beetles, fungus farming also occurs in another weevil subfamily, the Platypodinae [13,14]. Fungi are transmitted as spores in specialised cuticular pockets in ambrosia beetles, and are inoculated in the wood during the excavation of a new tunnel system in their host plant. They grow fine mycelia in the wood during egg laying [8]. After hatching, the larvae graze on a dense carpet of conidia which covers the tunnel walls. Ambrosia beetles are both functionally (food) and physiologically (hormones) dependent on these fungi. It has been documented that moulting and metamorphosis does not occur in the absence of fungal steroids [15]. It is therefore expected that a reversal to a wood based diet is not very likely once fungus farming has evolved.

The remaining species of Scolytinae mainly feed on bark and phloem, occasionally on seeds, and they take most of their nutrients from dead plant tissue excavated during tunnel construction [1]. These beetles are also associated with Ophiostomales fungi, which may provide food enrichment. Although it is not unusual that bark beetles have mycangia to facilitate transportation of fungal spores [8], they cannot make fungal gardens and have only a facultative association with fungi; hence, they can complete their life cycle without the addition of fungus to their diets. The fitness of certain bark beetle species (e.g. *Dendroctonus*) is nevertheless increased by addition of such fungi in their phloem diet [16,17]. This is a selective advantage for effective transmission. One can therefore readily imagine that a transition from a primarily bark- and phloem-based diet to a nutritional dependence on fungi is not a particularly difficult evolutionary event. Fungus farming has evolved multiple times in Scolytinae because of this nutritional advantage [13,18].

Multiple origins of fungus farming enable a meaningful comparison of the underlying ecological circumstances that may have spurred the transition from phloem to fungal feeding. Fungus farming beetles are found in wet tropical forests (with a few boreal exceptions), which is perhaps indicative of the ideal climate for ambrosia beetle species radiations. However, the ecological or climatic conditions under which fungus farming may have evolved and the timing of these feeding transitions have not been explored in detail.

Ambrosia beetles are also characterised by a generally gregarious feeding behaviour in the larvae, a behaviour that seems pre-adaptive for close inbreeding by sibling mating [19,20]. Regular inbreeding is the rule in several unrelated scolytine lineages and is characterised by female-biased offspring sex ratios. Fungus farming is potentially advantageous to developing sibling mating by congregating brood members in areas of fungal growth, which facilitates efficient mating between siblings. The direction of evolution from gregarious inbreeding to fungus farming or vice versa, or the assessment of whether or not these are completely unlinked phenomena, has not yet been examined in a phylogenetic context but see [1,8,19,21].

A detailed phylogenetic analysis of Scolytinae will enable us to test the sequence of the origin of fungus farming, gregarious feeding modes, and sibling mating. Hence, we have reconstructed the most comprehensively sampled phylogeny of Scolytinae to date, based on five genes for 200 Scolytinae species. This represents 123 of 250 genera in 24 of the currently 27 recognized tribes, including all known fungus farming lineages. Dates of evolutionary origin were estimated by calibrating molecular divergence rates using a significantly updated fossil record that set the minimum age of both Scolytinae and Curculioninae to more than 100 Ma [22-24].

## Results

**Figure 1:**
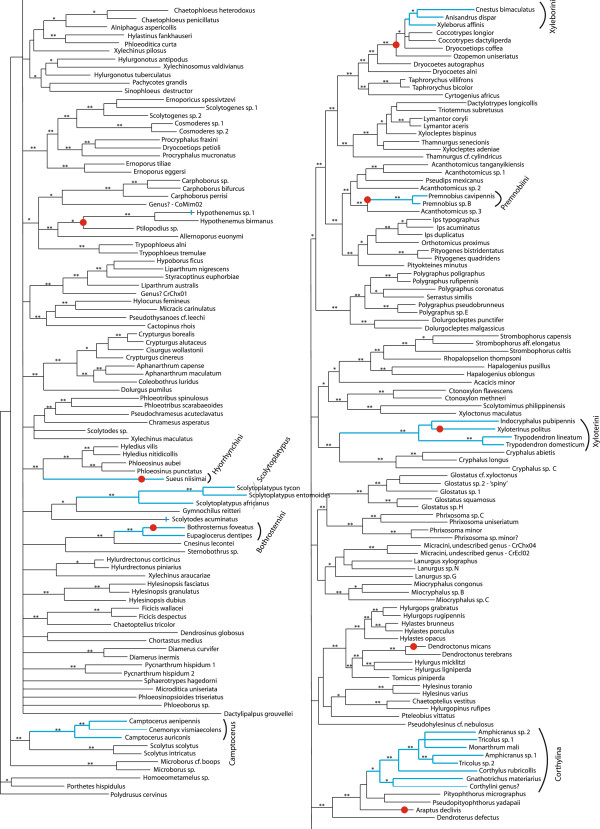
**Molecular phylogeny of Scolytinae.** Topology based on Bayesian analysis of 3,694 nucleotides from five unlinked gene fragments, using seven unlinked data partitions (mtDNA positions 1, 2, and 3, nucDNA protein encoding gene positions 1, 2, and 3, *28S* rRNA). Posterior probability marked on nodes by ** = 100, * = > 95. Obligate fungus farming is marked in blue, with blue hatch mark signifying ambrosia symbiosis in a single species. Red dots indicate the inferred origin of regular sibling mating.

All phylogenetic analyses revealed limited phylogenetic structure at deeper nodes, with most of the strongly supported nodes occurring at tribal and generic levels. Differences between the various Bayesian and parsimony analyses were not strongly supported and we have therefore used the Bayesian analysis based on seven partitions to illustrate the major topological findings in this report (Figure 1). Incongruent signal in the five independent markers was revealed by an overall negative support from *EF-1α* or *CAD* in the partitioned Bremer analyses (Additional file 1: Figure S1). However, re-analyses with exclusion of one or more partitions did not improve overall node support. Analyses of amino acid translated data from the four protein encoding genes combined resulted in minimal resolution, mainly with the ingroup separate from the outgroups, and with Scolytini subtending the most basal node in Scolytinae.

### Phylogenetic patterns

Eleven of the 24 included tribes (Additional file 2: Table S1) were monophyletic in all or some analyses, including Bothrosternini, Corthylini, Crypturgini, Phloeotribini, Phrixosomatini, Scolytoplatypodini, Scolytini, Xyleborini and Xyloterini (Hyorrhynchini and Cactopinini were represented by single species). Micracidini included Cactopinini and these taxa were monophyletic in some analyses (Figure 1). Dryocoetini was paraphyletic with respect to Xyleborini and Ipini was paraphyletic with respect to Premnobiini; these two clades formed well supported sister groups. Scolytoplatypodini was nested within parts of Hexacolini (genera *Scolytodes* and *Gymnochilus*). Several well-sampled tribes such as Hypoborini and Polygraphini were monophyletic with the exclusion of one genus each. The majority of conifer associated Hylurgini (previously Tomicini) formed two main clades – one southern hemisphere clade consisting of *Araucaria* associated species, and one northern clade associated with boreal Pinaceae. Although only weakly supported, Scolytini was monophyletic and subtended the Hexacolini genus *Microborus* and all other Scolytinae species.

### Timing and directionality of ambrosia feeding

Ten independent origins of fungus cultivation could be traced on the various topologies under accelerated parsimony optimisation (Figure 1), with eleven origins under delayed transformation. Two of these origins were the recent origins of single species in the genera *Hypothenemus* and *Scolytodes*. Hyorrhynchini was represented only by a single species (*Sueus niisimai*). The remaining seven groups of fungus farming beetles were monophyletic in all analyses, with the exception of *Camptocerus* that was sometimes paraphyletic with respect to *Cnemonyx*.

**Figure 2:**
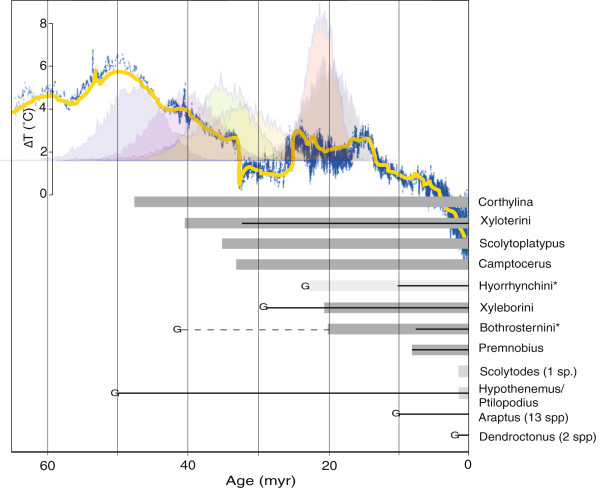
**Dates of origins of key evolutionary traits in Scolytinae.** Age of gregarious larval feeding (G), regular inbreeding by sibling mating (thin line), and ambrosia fungus feeding (grey box) in Scolytinae, based on crown ages as estimated in Beast (Analysis A). Stippled line and light grey indicate uncertainties associated with the lack of observations (Bothrosternini) or lack of phylogenetic sampling (Hyorrhynchini). Only those larvae that move freely and feed gregariously are considered truly ‘gregarious.’ Above, the Zachos curve showing variation in global temperature, and the posterior 95% distribution of crown age for Corthylina, Xyloterini, *Scolytoplatypus*, *Camptocerus*, Xyleborini, and the *Bothrosternus*-*Eupagiocerus* clade.

Minimum (crown) and maximum (stem) age estimates for ambrosia beetle clades are listed in Table 1. For the seven clades where crown age could be assessed, dates ranged from 8-48 Ma in analysis A (Figure 2; Additional file 3: Figure S2), with Corthylina as the likely earliest clade of fungus farming species. Xyloterini, Scolytoplatypodini and *Camptocerus* followed with ages of 40, 35 and 33 Ma. The origins of the *Bothrosternus* + *Eupagiocerus* and Xyleborini clades were younger, estimated to 20 and 21 Ma. The two different analyses provided very similar time estimates, with analysis B providing time estimates roughly 1-3 myr older (Table 1).

**Table 1:** Estimates of clade ages

	**Analysis A**		**Analysis B**	
**Clade**	**Crown age**	**Stem age**	**Crown age**	**Stem age**
*Bothrosternus* + *Eupagiocerus*	20,29 (14-27)	33,78 (40-56)	23.75 (15-34)	36.49 (27-45)
*Camptocerus*	33,40 (22-45)	35,88 (24-47)	34.94 (22-47)	38.41 (24-50)
Corthylina	48,03 (40-56)	50,33 (40-56)	49.62 (41-58)	52.61 (43-61)
Hyorrhynchini	-	69,78 (53-82)	-	71.62 (57-86)
*Hypothenemus curtipennis*	<1	-	<1	-
*Premnobius*	8,10 (5-12)	35,10 (27-45)	8.02 (5-12)	37.18 (28-46)
*Scolytodes unipunctatus*	<1	-	<1	-
*Scolytoplatypus*	35,25 (28-44)	53,22 (45-62)	36.60 (28-46)	55.74 (47-66)
Xyleborini	21,07 (17-25)	23,22 (19-28)	21.02 (17-25)	23.05 (19-28)
Xyloterini	40,58 (32-50)	68,26 (61-76)	41.90 (33-51)	70.36 (61-79)

Crown (min) and stem (max) ages were estimated from Beast analyses of all nucleotides, using (A) two fossil calibrations with oldest Scolytinae set to 100 Ma, and (B) three fossil calibrations with oldest Scolytinae set to 120 Ma (95% confidence intervals in brackets).

Each fungus farming lineage was sister to a bark feeding beetle lineage, with the exception of *Bothrosternus-Eupagiocerus* where the sister group *Cnesinus* consisted of pith feeders, and, in part, Xyleborini where the sister group *Coccotrypes* contained some seed feeding species (Table 2).

**Table 2:** Sister group contrasts of the fungus farming clade and its inferred closest relative

**Fungus cultivating clade**	**# spp**	**Sister lineage**	**# spp**	**Feeding mode (sister)**
Corthylina	**460**	*Pityophthorus*&*Pseudopityophthorus*	300	bark and leafstalks
*Camptocerus*	30	*Cnemonyx*	**50**	bark
*Bothrosternus* + *Eupagiocerus*	16	*Cnesinus*	**90**	pith, bark
Xyleborini	**1300**	*Coccotrypes*	120	Bark, seed and leafstalks
Xyloterini	24	*Cryphalus*	**200**	bark and leafstalks
*Scolytoplatypus*	**32**	*Gymnochilus*	15	bark
Hyorrhynchini	15	*Phloeosinus*&*Hyledius*	**100**	bark
*Premnobius*	25	*Acanthotomicus*	**50**	bark and leafstalks
*Hypothenemus curtipennis*	1	*Hypothenemus*	**120**	bark, seed and leafstalks
*Scolytodes unipunctatus*	1	*Scolytodes*	**150**	bark and leafstalks

Species numbers are from Wood & Bright [25]; the most diverse lineage is indicated in bold.

### The relative timing of gregarious feeding and inbreeding

The relative timing of ambrosia fungus cultivation, regular inbreeding and gregarious feeding modes are depicted in Figure 2. The oldest origins of regular inbreeding were estimated to be 50.1 Ma (analysis A) for *Hypothenemus* + *Ptilopodius*, and 29.3 Ma for the inbreeding Dryocoetini (*Ozopemon, Coccotrypes, Dryocoetiops*) plus Xyleborini. In clades where both inbreeding and ambrosia fungus farming occur (n = 6), inbreedingevolved first twice, fungus farming three times, and simultaneously once. Gregarious larval feeding occured in six clades and always occurred before or simultaneously with fungus farming (n = 4), and inbreeding (n = 6). Independence between fungus farming and gregarious feeding mode, and between fungus farming and regular inbreeding by sibling mating, was not rejected, while independence between gregarious feeding and sibling mating was rejected (Table 3). Transition rates indicated that fungus farming is more likely to evolve in groups with regular inbreeding or gregarious feeding mode, with the highest transition rates to fungus farming found in lineages with regular inbreeding.

**Table 3:** Correlation of fungus farming, gregariousness and regular inbreeding

**Comparison (X, Y)**	**Log likeli-hood diff.**	***P-*value**	**Q**_**12**_ **0,0 → 0,1**	**Q**_**13**_ **0,0 → 1,0**	**Q**_**24**_ **0,1 → 1,1**	**Q**_**34**_ **1,0 → 1,1**
Fungus farming – gregarious	4.74	0.12	0.195	0.182	**1.822**	0.001
Gregarious – fungus farming	4.48	0.12	0.115	0.178	0.001	**1.166**
Fungus farming – inbreeding	10.12	0.07	0.111	0.098	**4.091**	0.249
Inbreeding – fungus farming	10.11	0.06	0.098	0.111	0.248	**4.108**
Gregarious – inbreeding	11.17	0.05*	0.114	0.093	**4.279**	0.163
Inbreeding – gregarious	12.23	0.02*	0.097	0.172	0.520	**4.150**

Based on Pagel’s [26] test of independence between traits (100 simulations, 20 ML attempts). If Q_13 _is higher than Q_12_, X is more likely to evolve first, if Q_34_ is higher than Q_12_ then Y is more likely to depend on X, if Q_24_ is higher than Q_13_, then X is more likely to depend on Y; transition rates > 1 marked in bold.

## Discussion

Fungus farming has evolved at least ten times in Scolytinae, in contrast to the single origin of fungus farming in attine ants and macrotermitine termites [27,28]. Although limited resolution in tree topology was found in all types of analyses, the fungus farming taxa and sister lineages were well resolved. We found that all origins of fungus farming in Scolytinae were derived and a reversal to a non-fungal diet could not be traced on any of the tree topologies examined. It is noteworthy that although these findings are concordant with previous studies [18], our data are more complete in terms of taxon sampling, inclusive of a higher number of fungus farming taxa per clade. The only possible reversal indicated by some of the Bayesian topologies related to *Camptocerus* with respect to *Cnemonyx*. However, a more complete taxon sampling for these two genera showed that *Camptocerus* is indeed monophyletic [29]; Smith and Cognato, unpublished molecular data]. Our data thus corroborate the hypothesis that fungus farming is indeed a non-reversible evolutionary transition.

The many origins of fungus farming did not correlate strongly with some of the biological factors that benefit from a symbiotic relationship between fungi and beetles. Although we did observe a trend in fungus farming evolving more often in lineages with close inbreeding, the reverse transition rate (from outbreeding to inbreeding in fungus farming lineages) were negligibly low and the association between specific reproductive modes and fungus farming was not significantly correlated (see Table 3). This is perhaps most clearly illustrated by the complete lack of regular inbreeding in four ambrosia beetle lineages (*Scolytodes unipunctatus,* the genera *Camptocerus* and *Scolytoplatypus*, and the entire subtribe Corthylina). Furthermore is regular inbreeding the norm in true bark beetles such as in some *Dendroctonus* and *Araptus* [see e.g. [19]. Fungus farming is therefore a trait that at least sometimes evolves relatively independent of reproductive biology. Repeated origins of fungus farming must therefore be explained by additional ecological factors such as the frequent facultative association between bark beetles and fungi that grow in the phloem and bark of the host trees. Based on this perspective, it is notable that termites have only evolved fungus farming on one occasion even though fungus is an important food component for many other termite groups [27].

Compared with the timing of the origin of Scolytinae, more than 100 Ma, the development of obligate symbiotic fungus farming occurred relatively late. In all groups where an estimate of crown age was reliable, they revealed origins younger than 50 Ma, with 95% confidence interval ± 12 myr (Table 1). Xyloterini and Scolytoplatypodini had stem ages older than the crown age for Corthylina, which could potentially indicate a slightly older origin of fungus farming in these groups. However, it is equally likely that close relatives of these taxa were not included in our study which would overestimate the age of these fungus farmers. Regardless of these uncertainties, the Ophiostomales fungi have certainly existed much longer than the ambrosia beetles as shown by the multiple independent origins of the symbiotic fungi [18,30], and thus have likely been nutritionally advantageous to the early lineages of bark beetles that preceded the first ambrosia gardeners. In light of the ubiquitous presence of ambrosia beetles in pantropical forests, and the likely early availability of ambrosia fungi, one may wonder why such a successful adaptation should have taken so long to evolve. There are two particularly relevant factors that may not have been optimal at the earliest stage of bark beetle evolution – tropical forest diversity and climate.

About 98 percent of the known ambrosia beetle fauna is tropical or subtropical [25], which emphasizes that fungal symbiosis is largely dependent on moist conditions in warm climates [8,31]. Thus, the timing of modern moist tropical forests expansion may be relevant to the origin of fungus farming beetles. Elements of angiosperm-dominated tropical forests developed during the mid-Cretaceous, but did not radiate extensively until the Palaeocene or early Eocene era [32-35]. This time period experienced a thermal maximum (PETM) of some 5-8 degrees warmer climate from 58 to 45 Ma [36]. Several groups of animals and plants showed increased diversification associated with the increasing angiosperm dominance [37], in particular during or just after PETM [33,38-41] when tropical elements dominated floras and faunas from the equator to mid-latitudes e.g. [35,42,43]. Corthylina, Xyloterini, *Scolytoplatypus* and *Camptocerus* originated during or immediately after PETM and had likely taken advantage of the large tropical angiosperm forests emerging during this time period.

The only group of fungus cultivating insects that may have occurred in the Cretaceous period is a related group of weevils in the subfamily Platypodinae. Recent studies are inconsistent about the phylogenetic position of these beetles, but they are definitely part of the advanced weevil radiation [13,44,45]. Although the timing of this group seems problematic as a consequence of a generally higher substitution rate at independent genetic loci see [13], a late Cretaceous origin at 100-80 Ma seems realistic based on molecular data [13,45] and a fossil from Burmese amber (Grimaldi, pers. comm). Climate during this time period is less well understood, but was probably quite warm, dominated by Magnoliales and the early expanding Malphigiales [35,46]. However, the greatest part of the platypodine radiation took place much later, with more than 90 percent of the diversity originating in the Eocene and later time periods [13].

It is interesting that fungus farming in ants and termites have similarly late origins as in most Scolytinae beetles. Attine ants first originated around 50 Ma [12], similar to Corthylina beetles. However, the major radiation of these ants occurred later, around 20 Ma, which corresponds to our estimates for *Bothrosternus-Eupagiocerus* and the great Xyleborini radiation. During this intermediate ‘Antarctic thawing’ period [36], which lasted some 10 million years, tropical climates again dominated near mid-latitudes [35]. This is also the time period when the fungus gardening termites (Macrotermitinae) diversified [47], after their origin in tropical rainforests of Africa [48].

The late origin of the greatest ambrosia beetle radiation in Xyleborini is well supported by our data. Stem age was only 23 Ma (Additional file 3: Figure S2) for a clade that is closely related to bark beetles in the genera *Coccotrypes, Dryocoetiops* and *Ozopemon*[49-52]. The species diversity in Xyleborini is therefore unparalleled by any other ambrosia beetle lineage or other scolytine lineage. A recent origin of Xyleborini fits well with their absence from Dominican amber, a fossil source otherwise rich on older ambrosia beetle groups such as Corthylina and Platypodinae [53]. The great diversity of Xyleborini stands in contrast to a relatively modest diversity in the other clades of scolytine ambrosia beetles, particularly so in perspective of time. The reason for their great diversity is unclear. There are at least nine other scolytine clades of ambrosia beetles and only three of these are marginally more diverse than their sister group (see Table 2). Xyleborini are also characterized by regular inbreeding by sibling mating which is generally a great success factor in scolytine evolution, including *Hypothenemus* and related genera in Cryphalini. However, among the seven origins of regular inbreeding, only Xyleborini and inbreeding Cryphalini are more diverse than their sister group, so evidently there is no direct connection between inbreeding and diversification. There is therefore nothing overtly unique with this group of beetles compared with other ambrosia beetle lineages. To conclude, Xyleborini is most likely diverse because of chance effects, evolving at the right time (global warming in the ‘Antarctic thawing’ period) in the most productive parts of the globe, in the tropical regions.

## Conclusion

This study has provided the most accurate time estimate to date for the origin of fungus farming in bark and ambrosia beetles. Many origins occurred at different time periods, which resulted in a highly variable diversity for each lineage per time unit. However, they all shared a relatively late origin, which most likely occurred in periods of global warming and expanding wet tropical forests. Our taxonomic coverage was generally broad, which resulted in precise estimates for most origins of fungus farming. However, these estimates were less certain in Scolytoplatypodini, Xyloterini and Hyorrhynchini in which sister group relations were more distant. Additional research must aim at closing these taxonomic gaps.

The present study has furthermore demonstrated the challenge connected with resolving highly diverse taxon groups. Almost 4000 nucleotides were included from five independent molecular markers, but only the more derived clades were resolved, including all origins of fungus farming. Scolytinae is a very interesting group for many other kinds of evolutionary studies and detailed hypotheses may be tested if also deeper phylogenetic nodes obtain better resolution. As it currently stands, more protein encoding genes must be optimised to obtain such goals – a project of high priority in our laboratories.

## Methods

### Taxon sampling and data acquisition

Individuals used for DNA extraction and PCR are listed in Additional file 2: Table S1, inclusive of country of origin and genbank accession numbers. Three closely related outgroups were included. Platypodinae and *Coptonotus* were excluded because of their uncertain phylogenetic placement and the fact that Platypodinae has generally very long branch lengths which may influence the rooting at the base of Scolytinae [13].

Ambrosia beetles in the subfamily Scolytinae are defined as beetles actively cultivating fungi, and which differ from other bark and seed beetles by their exclusively fungal diet [8]. Inbreeding is defined as regular mating between siblings, with strongly female biased offspring sex ratio [19]. Gregarious feeding is defined by clustered siblings feeding and congregated development (without independent larval tunnels and pupal cradles), often forming variously rounded or cave-like structures in the wood. The character states for each included taxon are listed in Additional file 4: Table S2.

DNA extractions, PCR reactions, purifications and DNA sequencing reactions followed the protocols given in Jordal et al. [13].

### Alignment and phylogenetic analyses

All protein encoded gene sequences were aligned by eye, with introns identified by insertions demarcated by the initiating GT and terminating AG motif. These were excised before further analyses. Alignments of rDNA sequences from the D2-D3 domains of the large ribosomal subunit *28S* were initially aligned by the MUSCLE software [54] using default parameters, then re-aligned after pruning long expansion segments in *Phloeoborus* sp, *Diamerus curvifer*, *Dolurgocleptes punctifer* and *Dactylipalpus grouvellei*. The resulting Muscle alignment was adjusted slightly according to a secondary structure model for Scolytinae [55].

Phylogenetic trees were reconstructed in a Bayesian framework using Mr Bayes 3.1.2 [56] and by parsimony searches in PAUP* [57]. For the Bayesian analyses we selected the best model for each partition using Mr Model test [58]. We partitioned the nucleotide data by (a) genomes (2: mtDNA vs. nDNA) (b) genes (5: *COI, EF-1α, CAD, ArgK, 28S*), (c) positions per genome (7: 1st, 2nd and 3rd positions in mtDNA vs. 1st, 2nd and 3rd positions in the combined protein coding DNA data, and *28S*) and (d) gene specific positions (13: each codon position for each protein coding gene, and *28S*; see Additional file 5: Table S3). Most partitions had a GTR + I + Γ model selected by AIC, with only *COI* 3rd positions optimizing a GTR + Γ model (Additional file 5: Table S3). Amino acid translated data from the four protein encoding genes were subject to estimation of gene specific models (mixed evolutionary models). Fifty million generations were run on a Titan cluster of 8 CPUs hosted at http://www.bioportal.uio.no, with sampling every 1000 generation. The level of convergence from two parallel runs was inspected in live views of likelihoods for the two runs.

Parsimony analyses consisted of 2,000 heuristic searches with 30 random additions and TBR swapping for each search. Node support was estimated by 200 bootstrap replicates of 20 random addition replicates each. Gene specific contribution to each node was measured by partitioned Bremer support [59]. Ecological character transformation was traced in Mesquite [60] using the parsimony criterion, with test of correlation between fungus farming, gregariousness and inbreeding using Pagel’s [26] modified test of independence between traits. Statistical distributions were based on 20 maximum likelihood searches of 100 data simulations.

### Dating of nodes

We estimated divergence times in the software BEAST [61], with input files generated in the BEAST module BEAUti (Additional file 6: Table S4, Additional file 7: Table S5). Data were divided into five partitions consisting of mtDNA1 + 2, mtDNA3, nuc1 + 2, nuc3, *28S*. A Yule speciation process birth rate was implemented with a uniform distribution between 1 and 1,000. The Beast tree was calibrated by dating nodes with a relatively precise fossil date, hence using a normally distributed age for these calibration points (see below). Each analysis ran for 20 million generations, with a total of 4,000 trees sampled, deleting the first 2,000 trees as burn-in (Additional file 6: Table S4, Additional file 7: Table S5). The analyses were replicated once with the settings changed according to suggestions from BEAST: scale factor = 0.822 (default 0.75); window-size = 2.0 (default 1.0).

The oldest known Scolytinae fossil *Cylindrobrotus pectinatus* is possibly from early Aptian Lebanese amber [23]. This species cannot be assigned to any scolytine genus or tribe and likely is indicative of the most ancient form of Scolytinae, predating Scolytinae as currently defined [1]. Burmese amber includes the slightly younger (100 Ma) scolytine fossil in the extant genus *Microborus*[22] and thus indicates a more exact minimum age for Scolytinae. The fossil fauna of the advanced weevils is relatively young in this respect. The oldest fossil currently known is *Ararioerhinus* (Anthonomini) from the mid-Cretaceous Santana formation about 112-116 Ma [24]. Previous phylogenetic analyses of weevils has demonstrated an extremely narrow time window for the radiation of the advanced weevils, with Brachycerinae and Entiminae (here represented by *Polydrusus*) marginally older than the more advanced weevils such as Molytinae, Baridinae and Scolytinae [13,45,62]. Thus it is highly unlikely that Scolytinae and other advanced weevils are much older than the 120 myr indicated by the lower Aptian scolytine fossil.

Because there is a conflict between the age of the oldest scolytine and other advanced weevil fossils, two different analyses were made. In the first analysis (A) we used two fossil calibrations, including Scolytinae at 100 Ma (st. dev. = 1) and the advanced weevils at 112 Ma, excluding the Entiminae taxon *Polydrusus*. In the second analysis (B) we allowed for an older age of Scolytinae corresponding to the lower Aptian age of *Cylindrobrotus* at 120 Ma, the age of 100 Ma for the oldest node that includes *Microborus* (excluding the tribe Scolytini according to the amino acid coded data and supported by a previous study [13]), and we included the oldest known dryocoetine fossil that is likely ancestral to *Dryocoetes* (at the node subtending *D. alni*). The latter fossil was taken from Dominican amber with an approximate age of 30 Ma as one of very few reliable fossils that fits with our taxon sampling (see also Additional file 6: Table S4, Additional file 7: Table S5).

## Competing interests

The authors declare that they have no competing interests.

## Authors’ contributions

BHJ designed the study, collected and assembled beetle samples, carried out the molecular phylogenetic analyses and drafted the manuscript. AIC participated in the overall design of the study, collected some of the samples, and contributed to drafting the manuscript. Both authors have read and approved the final manuscript.

## Supplementary Material

Additional file 1 **Figure S1.** Partitioned Bremer support values for each of the five gene fragments included. Their relative support was estimated on two tree topologies: the 84-taxa parsimony topology and the Bayesian 7-partitions topology pruned to 84 taxa. Click here for file

Additional file 2 **Table S1.** Taxon sampling and their respective accession numbers in GenBank (missing data denoted by ’-’). Click here for file

Additional file 3 **Figure S2.** Beast tree topology showing each origin of fungus farming in thick blue branches, and each origin of regular inbreeding marked by a black dot. Below is the Zachos curve of temperature variation during the Cenozoic. Click here for file

Additional file 4 **Table S2.** Character states for the taxa sampled, with the absence (0) or presence (1) of fungus farming, gregarious larval feeding and regular inbreeding. Click here for file

Additional file 5 **Table S3.** Properties of each partition, estimated from 84 taxa with no missing data. Click here for file

Additional file 6 **Table S4.** Xml file generated by Beauti and analyzed in Beast (analysis A). Click here for file

Additional file 7 **Table S5.** Xml file generated by Beauti and analyzed in Beast (analysis B). Click here for file
