# Supplementary figures and images for: Molecular phylogeny of bark and ambrosia beetles reveals multiple origins of fungus farming during periods of global warming
Source: BMC Evol Biol. 2012 Aug 1;12:133. doi: 10.1186/1471-2148-12-133 (PMC3514184; doi:10.1186/1471-2148-12-133)

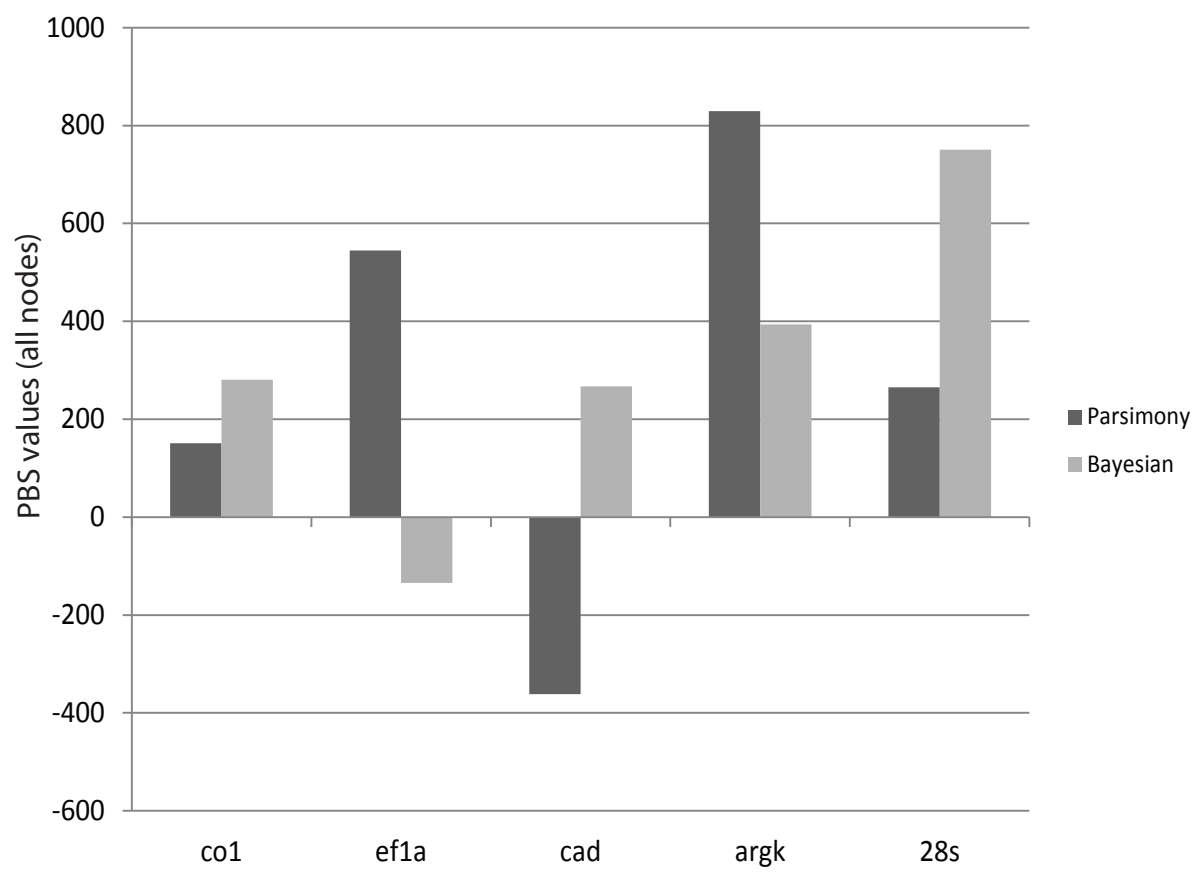

Supplement: Additional file 1 — Figure S1. Partitioned Bremer support values for each of the five gene fragments included. Their relative support was estimated on two tree topologies: the 84-taxa parsimony topology and the Bayesian 7-partitions topology pruned to 84 taxa. [file 1471-2148-12-133-S1.pdf]

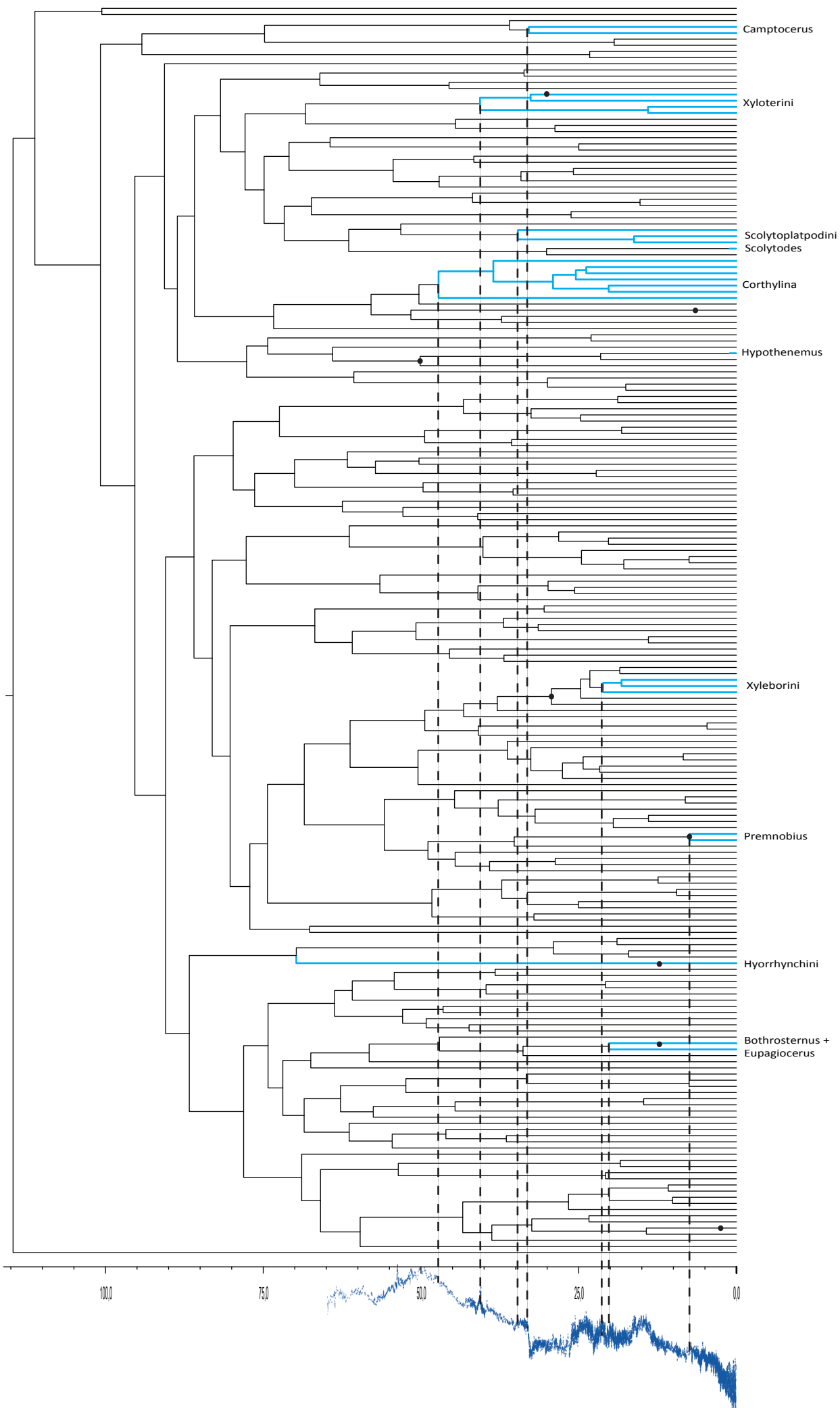

Supplement: Additional file 3 — Figure S2. Beast tree topology showing each origin of fungus farming in thick blue branches, and each origin of regular inbreeding marked by a black dot. Below is the Zachos curve of temperature variation during the Cenozoic. [file 1471-2148-12-133-S3.pdf]
