# Supplementary material for: Molecular phylogeny of bark and ambrosia beetles reveals multiple origins of fungus farming during periods of global warming
Source: BMC Evol Biol. 2012 Aug 1;12:133. doi: 10.1186/1471-2148-12-133 (PMC3514184; doi:10.1186/1471-2148-12-133)
Supplement: Additional file 2 — Table S1. Taxon sampling and their respective accession numbers in GenBank (missing data denoted by ’-’). [file 1471-2148-12-133-S2.docx]

**Supplementary Table S1.** Taxon sampling and their respective accession numbers in GenBank (missing data denoted by '-')

| CODE | TRIBE | SPECIES | COUNTRY | LOCALITY | **CO1** | **EF-1a** | **28S** | **CAD** | **ArgK** |
| --- | --- | --- | --- | --- | --- | --- | --- | --- | --- |
| EnPol01 | Entiminae | Polydrusus cervinus | Norway | Telemark, Gvarv | HQ883729 | HQ883729 | HQ883568 | HQ883793 | HQ883884 |
| CsXxA01 | Conoderinae | Homoeometamlus spA | Uganda | Kibale | HQ883643 | HQ883723 | HQ883558 | HQ883785 | HQ883872 |
| MoPor01 | Molytinae | Porthetes hispidus | South Africa | EC, Kokstad | HQ883666 | HQ883737 | HQ883577 | HQ883805 | HQ883895 |
| BoBot01 | Bothrosternini | Bothrosternus foveatus | Costa Rica | Birri | - | JX264073 | JX263669 | JX263984 | JX263875 |
| BoCne01 | Bothrosternini | Cesinus lecontei | Costa Rica | Birri | JX263780 | AF308397 | AF308352 | JX263985 | JX263876 |
| BoEup01 | Bothrosternini | Eupagiocerus dentipes | Costa Rica | Birri | JX263781 | JX264074 | JX263670 | JX263986 | JX263877 |
| BoSte01 | Bothrosternini | Sternobothrus sp. | Brasil | Esperito Santo | JX263782 | - | JX263671 | JX263987 | - |
| CaCac01 | Cactopinini | Cactopinus rhois | USA, CA | Riverside Co., San Bernadino NF | JX263783 | JX264075 | EU090343 | - | JX263878 |
| CoAmp01 | Corthylini | Amphicranus sp. | Costa Rica | 11 km SE La Virgen | JX263784 | JX264076 | JX263676 | JX263993 | JX263883 |
| CoAmp02 | Corthylini | Amphicranus sp. B | Costa Rica |  | JX263785 | JX264077 | JX263677 | JX263994 | JX263884 |
| CoAra01 | Corthylini | Araptus declivis | Argentina | Agua Blanca, Bolivian border | AF187123 | AF186671 | AF375297 | JX263995 | - |
| CoCor01 | Corthylini | Corthylus rubricollis | Costa Rica |  | JX263786 | JX264078 | JX263678 | JX263996 | JX263885 |
| CoDen01 | Corthylini | Dendroterus defectus | Panama |  | JX263787 | JX264079 | JX263679 | JX263997 | JX263886 |
| CoMim02 | Corthylini | Genus? | Tanzania | Mang'ula: Udz. HQ | JX263789 | JX264081 | - | JX263999 | JX263888 |
| CoXxx01 | Corthylini | Genus? | Costa Rica |  | - | JX264083 | - | JX264004 | JX263892 |
| CoGna01 | Corthylini | Gnathotrichus materiarius | USA | NC, Pisgah Nat. Forest | JX263788 | JX264080 | JX263680 | JX263998 | JX263887 |
| CoMon01 | Corthylini | Monarthrum mali | USA | NC, Pisgah Nat. Forest | JX263790 | - | JX263681 | JX264000 | - |
| CoPit01 | Corthylini | Pityophtorus micrographus | Norway | Trondheim | EU191840 | EU191872 | JX263682 | JX264001 | JX263889 |
| CoPsp01 | Corthylini | Pseudopityophthorus yavapaii | USA | AZ, S. Tucson | - | AF375271 | AF375305 | - | - |
| CoTri01 | Corthylini | Tricolus sp.A | Costa Rica | Futerpe | - | JX264082 | JX263683 | JX264002 | JX263890 |
| CoTri02 | Corthylini | Tricolus sp.B | Costa Rica |  | JX263791 | - | JX263684 | JX264003 | JX263891 |
| CrAll01 | Cryphalini | Allernoporus euonymi | Russia | Primorsky Krai, Anisimovka | - | JX264084 | JX263685 | JX264005 | JX263893 |
| CrPti01 | Cryphalini | Cosmoderes sp. 1 | Papua New Guinea | Asiki Road, Bulolo | - | JX264095 | JX263698 | JX264015 | JX263906 |
| CrCos01 | Cryphalini | Cosmoderes sp. 2 | Papua New Guinea | Wau, Mt. Kaindi | JX263794 | JX264087 | JX263687 | JX264007 | JX263896 |
| CrCry02 | Cryphalini | Cryphalus abietis | Sweden | Klöverhult, Mönsterås | JX263795 | - | JX263688 | - | JX263897 |
| CrCry04 | Cryphalini | Cryphalus longulus | Russia | Primorsky Krai, 20km w Andreev | JX263796 | JX264088 | JX263689 | JX264008 | JX263898 |
| CrEcl01 | Cryphalini | Cryphalus sp. C | Madagascar |  | JX263798 | - | JX263691 | - | JX263900 |
| CrErn04 | Cryphalini | Ernoporicus spessivtzevi | Russia | Primorsky Krai, Anisimovka | JX263800 | JX264091 | JX263694 | JX264011 | JX263901 |
| CrErn05 | Cryphalini | Ernoporus eggersi | Russia | Primorsky Krai, Vladivostok | JX263801 | - | - | JX264012 | JX263902 |
| CrErn03 | Cryphalini | Ernoporus tiliae | Czeck Rep. | Moravia,Breclav, Kanci obora | EU191841 | EU191873 | JX263693 | JX264010 | - |
| CrHyp02 | Cryphalini | Hypothenemus birmanus | Thailand | Rai Lai Beach | JX263803 | JX264093 | JX263696 | - | JX263904 |
| CrHyp01 | Cryphalini | Hypothenemus cf. eruditus | USA | NC, Old Fort, Curtis Creek Rd. | JX263802 | JX264092 | JX263695 | JX264013 | JX263903 |
| CrCry05 | Cryphalini | Procryphalus fraxini | Russia | Primorsky Krai, Anisimovka | JX263797 | JX264089 | JX263690 | JX264009 | JX263899 |
| CrPro01 | Cryphalini | Procryphalus mucronatus | USA | Utah: Alta Canyon, SLC | JX263804 | JX264094 | JX263697 | JX264014 | JX263905 |
| CrPti02 | Cryphalini | Ptilopodius sp. | Sarawak | Lambir NP | - | - | JX263699 | - | - |
| CrXxx02 | Cryphalini | Scolytogenes nr onyanganus | Uganda | Kibale Forest | - | AF375270 | - | - | - |
| CrXxx01 | Cryphalini | Scolytogenes sp. | South Africa | EC: Alexandria forest | - | JX264098 | - | - | - |
| CrTry01 | Cryphalini | Trypophloeus alni | Russia | St. Petersburg | JX263805 | JX264096 | - | JX264016 | - |
| CrTry04 | Cryphalini | Trypophloeus tremulae | Ukraine | Crimea | - | JX264097 | - | JX264017 | JX263907 |
| CgAph02 | Crypturgini | Aphanarthrum capense | South Africa | EC: Ecca Pass, N. Grahamstown | EU143701 | EU143705 | JX263672 | JX263988 | JX263879 |
| CgCol01 | Crypturgini | Aphanarthrum maculatum | South Africa | EC: Ecca Pass, N. Grahamstown | EU143703 | EU143707 | JX263673 | JX263989 | JX263880 |
| CgCis01 | Crypturgini | Cisurgus wollastoni | El Hierro |  | AY514985 | AY500981 | - | - | - |
| CgCol03 | Crypturgini | Coleobothrus luridus | Gran Canaria |  | AY514989 | AY500985 | JX263674 | - | JX263881 |
| CgCryAlu | Crypturgini | Crypturgus alutaceus | USA |  | EU011815 | EU011827 | EU090328 | - | - |
| CgCry02 | Crypturgini | Crypturgus borealis | Canada |  | AF187130 | AY500991 | JX263675 | JX263991 | JX263882 |
| CgCry01 | Crypturgini | Crypturgus cinereus | Russia | St. Petersburg | EU011803 | EU011816 | - | JX263990 | - |
| CgDol01 | Crypturgini | Dolurgus pumilus | USA |  | AY514904 | AY500900 | - | JX263992 | - |
| DiAca01 | Diamerini | Acacicis minor | Queensland | Gympie | AY377000 | JX264099 | JX263701 | JX264019 | JX263908 |
| DiDia01 | Diamerini | Diamerus curvifer | Papua New Guinea | Madang | JX263807 | JX264100 | EU090348 | - | - |
| DiDia03 | Diamerini | Diamerus inermis | Tanzania | Mang'ula: Udz. HQ | JX263808 | JX264101 | JX263702 | JX264020 | JX263909 |
| DiSph01 | Diamerini | Sphaerotrypes hagedorni | Cameroon | Limbe Bot. Garden | JX263809 | JX264102 | JX263703 | JX264021 | JX263910 |
| DiStr02 | Diamerini | Strombophorus capensis | South Africa | EC: Hogsback | EU191845 | EU191877 | - | - | - |
| DiStr03 | Diamerini | Strombophorus celtis | Uganda | Kibale Forest | - | AF308400 | AF308355 | - | - |
| DiStr04 | Diamerini | Strombophorus cf. elongatus | Tanzania | Mang'ula: Udz. HQ | JX263810 | JX264103 | JX263704 | JX264022 | JX263911 |
| DrCoc01 | Dryocoetini | Coccotrypes dactyliperda | Argentina |  | AF444064 | AF444078 | GU808611 | GU808649 | GU808686 |
| DrCoc00 | Dryocoetini | Coccotrypes longior | Thailand |  | AF438515 | AF259871 | GU808612 | GU808650 | - |
| DrCyr01 | Dryocoetini | Cyrtogenius africus | South Africa | EC: Ecca Pass, N. Grahamstown | JX263811 | JX264104 | JX263705 | JX264023 | JX263912 |
| DrDac01 | Dryocoetini | Dactylotrypes longicollis | Spain | Tenerife | AF187119 | AF186667 | JX263709 | - | - |
| DrDry02 | Dryocoetini | Dryocoetes alni | Norway | Byneset, Trondheim | AF438508 | AF439742 | - | - | JX263918 |
| DrDry01 | Dryocoetini | Dryocoetes autographus | Russia | St. Petersburg | JX263816 | JX264109 | HQ883565 | HQ883791 | HQ883880 |
| DrDrc02 | Dryocoetini | Dryocoetiops coffea | Papua New Guinea | Lae | AF187122 | AF186670 | - | - | - |
| DrDrc01 | Dryocoetini | Dryocoetiops petioli | Thailand | Chiang Mai | JX263815 | JX264108 | JX263711 | JX264028 | JX263917 |
| DrLym02 | Dryocoetini | Lymantor aceris | Russia | Primorsky Krai, 20km w Andreev | JX263817 | JX264110 | JX263713 | JX264030 | JX263920 |
| DrLym01 | Dryocoetini | Lymantor coryli | Sweden | Oskarshamn | AF438516 | AF439743 | JX263712 | JX264029 | JX263919 |
| DrOzo02 | Dryocoetini | Ozopemon uniseriatus | Papua New Guinea | Asiki Road, Bulolo | AF438506 | AF439740 | JX263714 | JX264031 | JX263921 |
| DrTap02 | Dryocoetini | Taphrorychus bicolor | Norway | Vanvik, Sauda | AF187115 | AF186663 | JX263716 | - | JX263923 |
| DrTap01 | Dryocoetini | Taphrorychus villifrons | Ukraine | Crimea | JX263818 | JX264111 | JX263715 | - | JX263922 |
| DrCyr03 | Dryocoetini | Thamnurgus cylindricus | Cameroon | Mt. Cameroon, slopes | JX263813 | JX264106 | JX263707 | JX264025 | JX263914 |
| DrThm01 | Dryocoetini | Thamnurgus senicionis | Uganda | Mt. Elgon | AF187114 | AF186662 | JX263717 | JX264032 | JX263924 |
| DrTri01 | Dryocoetini | Triotemnus subretusus | Spain | El Hierro | EU191868 | EU191900 | - | JX264033 | JX263925 |
| DrXyl03 | Dryocoetini | Xylocleptes adeniae | Tanzania | Udzungwa, Sanje trail, 1000 m alt | JX263819 | JX264112 | - | JX264034 | JX263926 |
| DrXyl01 | Dryocoetini | Xylocleptes bispinus | Ukraine | Crimea | EU191848 | EU191880 | EU090347 | HQ883792 | HQ883881 |
| CtGym01 | Hexacolini | Gymnochilus reitteri | Costa Rica | Castilla | HQ883644 | - | EU090353 | HQ883786 | HQ883873 |
| CtMic01 | Hexacolini | Microborus cf. boops | Madagascar |  | HQ883645 | HQ883724 | HQ883559 | HQ883787 | - |
| CtMic03 | Hexacolini | Microborus sp. | Cameroon | Mt. Cameroon | HQ883646 | - | HQ883560 | HQ883788 | HQ883874 |
| CtPyc01 | Hexacolini | Pycnarthrum hispidum 1 | Costa Rica | Castilla | HQ883647 | - | EU090352 | HQ883789 | HQ883875 |
| CtPyc012 | Hexacolini | Pycnarthrum hispidum 2 | Mexico |  | HQ883648 | - | HQ883561 | - | HQ883876 |
| CtSct01 | Hexacolini | Scolytodes acuminatus | Costa Rica |  | EU191844 | EU191876 | EU090351 | HQ883790 | HQ883877 |
| CtSct04 | Hexacolini | Scolytodes sp. | Peru | Perene River | JX263806 | - | - | JX264018 | - |
| HtHyt02 | Hylastini | Hylastes brunneus | Norway | Op: Bøverdalen, Lom | JX263831 | JX264120 | - | JX264046 | - |
| HtHyt05 | Hylastini | Hylastes opacus | Sweden | Gotland | HQ883660 | HQ883732 | HQ883927 | HQ883799 | JX263937 |
| HtHyt08 | Hylastini | Hylastes porculus | USA | NC, Pisgah Nat. Forest | AF375321 | AF308430 | AF308387 | JX264047 | JX263938 |
| HtHyg02 | Hylastini | Hylurgops glabratus | Norway | NTr: Øksenøy, Bindalen | JX263830 | JX264119 | JX263728 | JX264045 | JX263936 |
| HtHyg09 | Hylastini | Hylurgops rugipennis | USA | NC, Pisgah Nat. Forest | HQ883659 | AF308408 | AF308408 | HQ883798 | HQ883889 |
| HlAln01 | Hylesinini | Alniphagus aspericollis | USA | Washington state | EU191849 | EU191881 | AF308367 | HQ883794 | HQ883885 |
| HlDac01 | Hylesinini | Dactylipalpus grouvellei | Ghana | Bokuro-Abaa | HQ883656 | HQ883731 | HQ883570 | HQ883795 | HQ883886 |
| HlFic02 | Hylesinini | Ficicis despectus | Papua New Guinea | Madang, Beitata | AY376999 | AY377063 | JX263719 | JX264036 | JX263928 |
| HlFic01 | Hylesinini | Ficicis wallacei | Papua New Guinea | Madang, Beitata | JX263821 | JX264113 | JX263718 | JX264035 | JX263927 |
| HlHap02 | Hylesinini | Hapalogenius oblongus | Uganda | Kibale Forest, S. Fort Portal | JX263823 | AF308412 | JX263721 | JX264038 | JX263930 |
| HlHap01 | Hylesinini | Hapalogenius pusillus | South Africa | WC: Knysna, Gouna | JX263822 | - | JX263720 | JX264037 | JX263929 |
| HlHlt02 | Hylesinini | Hylastini fankhauseri | Austria | Vienna | JX263824 | JX264114 | JX263722 | JX264039 | JX263931 |
| HlHnp03 | Hylesinini | Hylesinopsis dubius | Uganda | Budongo, N. Masindi | JX263827 | AF308401 | AF308356 | - | JX263934 |
| HlHnp01 | Hylesinini | Hylesinopsis fasciatus | Cameroon | Mt. Cameroon, S lope | JX263825 | JX264115 | JX263723 | JX264040 | JX263932 |
| HlHnp02 | Hylesinini | Hylesinopsis granulatus | Cameroon | Mt. Cameroon, S lope | JX263826 | JX264116 | JX263724 | JX264041 | JX263933 |
| HlHyl01 | Hylesinini | Hylesinus toranio | England | Norwich | JX263828 | JX264117 | JX263725 | JX264042 | - |
| HlHyl02 | Hylesinini | Hylesinus varius | Sweden | Gotland, E. Visby | HQ883657 | AF308409 | AF308365 | HQ883796 | HQ883887 |
| HlPhb02 | Hylesinini | Phloeoborus sp. | Guyana | Iwokrama | HQ883658 | - | HQ883571 | HQ883797 | HQ883888 |
| HlPte02 | Hylesinini | Pteleobius vittatus | Czeck Rep. | Bacov, Velky Osek | JX263829 | JX264118 | JX263726 | JX264043 | JX263935 |
| HlRho01 | Hylesinini | Rhopalopselion thompsoni | Ghana | Ankasa | EU191850 | EU191882 | JX263727 | JX264044 | - |
| ToCha02 | Hylurgini | Chaetoptelius tricolor | Queensland | Bunya Mts. | JX263863 | JX264144 | JX263765 | - | JX263968 |
| ToCha01 | Hylurgini | Chaetoptelius vestitus | Morocco | Agadir, north | JX263862 | JX264143 | - | JX264067 | JX263967 |
| ToDen01 | Hylurgini | Dendroctonus micans | Estonia | Rohukula, Haapsalu | HQ883680 | HQ883749 | HQ883591 | HQ883824 | JX263969 |
| ToDen02 | Hylurgini | Dendroctonus terebrans | USA | Georgia State | AF375315 | AF308429 | AF308386 | - | - |
| ToHdr01 | Hylurgini | Hylurdrectonus corticinus | Papua New Guinea | PNG, Wau, State Forest | AY040289 | AY040316 | JX263766 | - | JX263970 |
| ToHdr02 | Hylurgini | Hylurdrectonus pinarius | Australia | Australia, Qsld., Yarraman state Forest | AY040290 | AY040317 | JX263767 | - | JX263971 |
| ToHgn01 | Hylurgini | Hylurgonotus antipodus | Argentina |  | AY040291 | AF308419 | AF308376 | - | JX263972 |
| ToHgn02 | Hylurgini | Hylurgonotus tuberculatus | Argentina |  | AF375313 | - | AF308375 | - | JX263973 |
| ToHrg01 | Hylurgini | Hylurgopinus rufipes | Canada | Winnipeg, Manitoba | JX263864 | JX264145 | JX263768 | JX264068 | JX263974 |
| ToHyr01 | Hylurgini | Hylurgus ligniperda | New Zealand | Woodhill Forest, Auckland | AY040292 | JX264146 | JX263769 | - | JX263975 |
| ToHyr02 | Hylurgini | Hylurgus micklitzi | Spain |  | AY040293 | - | JX263770 | - | - |
| ToPac01 | Hylurgini | Pachycotes grandis | New Caledonia |  | JX263865 | - | JX263771 | - | - |
| ToPsh01 | Hylurgini | Pseudohylesinus nebulosus | USA | Utah: Beaver Canyon | AF375316 | AF308422 | AF308379 | - | - |
| ToSin01 | Hylurgini | Sinophloeus destructor | Argentina | Neuquen | AF375314 | AF308420 | AF308377 | - | JX263976 |
| ToTom01 | Hylurgini | Tomicus piniperda | Norway | Lom, Elveseter | HQ883681 | HQ883750 | HQ883592 | HQ883825 | HQ883911 |
| ToXch01 | Hylurgini | Xylechinosomus valdivianus | Argentina | Neuquen | AF375312 | AF308366 | AF308410 | - | - |
| ToXyl02 | Hylurgini | Xylechinus araucariae | New Caledonia |  | JX263867 | JX264147 | - | - | **-** |
| PtPht02 | Hylurgini | Xylechinus maculatus | Argentina | Neuquen | JX263856 | JX264138 | JX263758 | - | JX263961 |
| ToXyl01 | Hylurgini | Xylechinus pilosus | Sweden | Tärnaby | JX263866 | - | JX263772 | JX264069 | JX263977 |
| HcSue01 | Hyorrhynchini | Sueus niisimai | Singapore | Bhukit Timah | JX263820 | AF308399 | AF308354 | - | - |
| HyCha01 | Hypoborini | Chaetophloeus heterodoxus | USA | Utah: Alta | JX263832 | - | JX263729 | - | - |
| HyCha02 | Hypoborini | Chaetophloeus penicillatus | USA | Arizona | - | AF308415 | AF308371 | - | - |
| CrCh?01 | Hypoborini | Genus? | Madagascar |  | JX263792 | JX264085 | - | JX264006 | JX263894 |
| HyHyb01 | Hypoborini | Hypoborus ficus | Greece | Naxos | AY377006 | AY377070 | EU090350 | - | JX263939 |
| HyLip01 | Hypoborini | Liparthrum australis | South Africa | WC: Knysna, Gouna | EU191851 | EU191883 | - | - | - |
| HyLip02 | Hypoborini | Liparthrum nigrescens | Gran Canaria | Tenerife+Gran Canaria | AY377032 | AY377096 | AF308370 | - | - |
| HySty01 | Hypoborini | Styracoptinus euphorbiae | South Africa | EC: Ecca Pass, N. Grahamstown | EU191852 | EU191884 | JX263730 | - | - |
| IpAca01 | Ipini | Acanthotomicus sp. 1 | Cameroon | Bomana falls | JX263833 | JX264121 | JX263731 | JX264048 | JX263940 |
| DrCyr02 | Ipini | Acanthotomicus sp. 2 | Cameroon | Mt. Cameroon, Mann Spring | JX263812 | JX264105 | JX263706 | JX264024 | JX263913 |
| DrCyr04 | Ipini | Acanthotomicus sp. 3 | Cameroon | Bakingili, W. Limbe | JX263814 | JX264107 | JX263708 | JX264026 | JX263915 |
| IpAca02 | Ipini | Acanthotomicus tanganyikiensis | Uganda | Kibale Forest | AF187126 | AF186674 | JX263732 | - | - |
| IpIps02 | Ipini | Ips acuminatus | Norway | Hjuksebø, Notodden | HQ883661 | HQ883733 | HQ883573 | HQ883801 | HQ883891 |
| IpIps03 | Ipini | Ips duplicatus | Estonia | Mooste, Põlva | JX263834 | JX264122 | JX263733 | JX264050 | JX263942 |
| IpIps01 | Ipini | Ips typographus | Estonia | Mooste, Põlva | EU191853 | EU191885 | - | JX264049 | JX263941 |
| IpOrt01 | Ipini | Orthotomicus proximus | Sweden | Salsån, Svenstavik | JX263835 | JX264123 | JX263734 | JX264051 | JX263943 |
| IpPit01 | Ipini | Pityogenes bistridentatus | Ukraine | Crimea, Yalta | HQ883662 | HQ883734 | HQ883573 | HQ883801 | HQ883891 |
| IpPit03 | Ipini | Pityogenes quadridens | Sweden | Gotland | JX263836 | JX264124 | JX263735 | - | JX263944 |
| IpPtk01 | Ipini | Pityokteinus minutus | USA |  | AF187125 | AF186673 | - | - | - |
| IpPse01 | Ipini | Pseudips mexicanus | USA |  | - | AF397649 | EU090299 | - | - |
| CrCh?04 | Micracidini | Genus? | Madagascar |  | JX263793 | JX264086 | JX263686 | - | JX263895 |
| CrEcl02 | Micracidini | Genus? | Madagascar |  | JX263799 | JX264090 | JX263692 | - | - |
| MiHyl01 | Micracidini | Hylocurus femineus | USA | AZ, Madera Canyon, S. Tucson | AF187108 | AF186678 | JX263736 | JX264052 | - |
| MiLan02 | Micracidini | Lanurgus sp. G | South Africa | WC: Nature's Valley | JX263837 | JX264125 | - | - | - |
| MiTra01 | Micracidini | Lanurgus sp. N | South Africa | WC: Nature's Valley | JX263842 | JX264127 | JX263741 | JX264055 | - |
| MiLan01 | Micracidini | Lanurgus xylographus | South Africa | EC: Stutterheim, Kologha forest | EU191855 | EU191887 | - | - | - |
| MiMic01 | Micracidini | Micracis carinulatus | USA | AZ, Herb Martyr, S. Tucson | AF187107 | AF186677 | AF375303 | - | - |
| MiMio02 | Micracidini | Miocryphalus congonus | Cameroon | Limbe, Ekonjo | JX263839 | - | JX263738 | JX264053 | JX263945 |
| MiMio03 | Micracidini | Miocryphalus sp. B | Cameroon | Mt. Cameroon, Mann Spring | JX263840 | - | JX263739 | JX264054 | JX263946 |
| MiMio01 | Micracidini | Miocryphalus sp. C | South Africa | EC: Hogsback | JX263838 | - | JX263737 | - | - |
| MiPse01 | Micracidini | Pseudothysanoes cf. leechi | USA | California, Hastings Reserve | JX263841 | JX264126 | JX263740 | - | - |
| PhChr01 | Phloeosinini | Chramesus asperatus | USA | AZ: S. Tucson | JX263843 | AF308406 | AF308362 | JX264056 | JX263947 |
| PhDen01 | Phloeosinini | Dendrosinus globosus | Peru | Juanin Prov., P. Ocopa | JX263844 | - | JX263742 | - | - |
| PhHyl02 | Phloeosinini | Hyledius nitidicollis | Malaysia | Sabah: Danum Valley | EU191856 | EU191888 | JX263744 | - | JX263948 |
| PhHyl01 | Phloeosinini | Hyledius vilis | Sarawak | Bako | JX263845 | JX264128 | JX263743 | - | - |
| PtNew01 | Phloeosinini | Microditica uniseriata | Thailand |  | GQ470890 | GQ470891 | JX263757 | - | - |
| PhPhd01 | Phloeosinini | Phloeoditica curta | Bangladesh | Sunderbaans | GQ470889 | AF308402 | JX263745 | - | JX263949 |
| PhPps01 | Phloeosinini | Phloeosinopsioides formosanus | Papua New Guinea | Wau Ecol St. | JX263847 | JX264130 | JX263747 | JX264057 | JX263951 |
| PhPhl01 | Phloeosinini | Phloeosinus aubei | Ukraine | Crimea | JX263846 | JX264129 | JX263746 | - | JX263950 |
| PhPhl03 | Phloeosinini | Phloeosinus punctatus | USA | WA | HQ883668 | AF308405 | AF308361 | HQ883808 | HQ883898 |
| PhPch01 | Phloeosinini | Pseudochramesus acuteclavatus | Argentina | Salta, 7 km SW Gen. Enrique Mosconi | AF375328 | AF308404 | AF308360 | HQ883807 | HQ883897 |
| PtPht05 | Phloeotribini | Phloeotribus scarabaeoides | Spain | Andalusia, 5 km from Berja | EU191863 | EU191895 | JX263759 | JX264064 | JX263962 |
| PtPht01 | Phloeotribini | Phloeotribus spinulosus | Norway | Lierne | EU191862 | EU191894 | HQ883585 | HQ883816 | HQ883905 |
| PxGen01 | Phrixosomini | Phrixosoma concavifrons | Tanzania | Udzungwa, Sanje trail, 1400 m alt | JX263857 | JX264139 | JX263760 | - | JX263963 |
| PxPrx01 | Phrixosomini | Phrixosoma minor | Costa Rica |  | JX263858 | JX264140 | JX263761 | - | JX263964 |
| PxPrx03 | Phrixosomini | Phrixosoma sp. ?minor | Peru | Loreto Prov., 60km SW Iquito | JX263860 | - | JX263763 | JX264066 | JX263966 |
| PxPrx02 | Phrixosomini | Phrixosoma uniseriatum | Cameroon | Limbe, Ekonjo | JX263859 | JX264141 | JX263762 | JX264065 | JX263965 |
| PoCar03 | Polygraphini | Carphoborus bifurcus | USA | FL: Bay Co, Panama City | EU191859 | EU191891 | - | - | - |
| PoCar01 | Polygraphini | Carphoborus perrisi | Morocco | Agadir, north | EU191857 | EU191889 | JX263748 | JX264058 | JX263952 |
| PoCar02 | Polygraphini | Carphoborus sp. | USA | Calif: Riverside Co, James Reserve | EU191858 | EU191890 | JX263749 | - | JX263953 |
| PoCho01 | Polygraphini | Chortastus medius | Cameroon | Limbe, Ekonjo | JX263848 | JX264131 | JX263750 | JX264059 | JX263954 |
| DrDol02 | Polygraphini | Dolurgocleptes malgassicus | Madagascar |  | EU191847 | EU191879 | - | - | - |
| DrDol01 | Polygraphini | Dolurgocleptes punctifer | Madagascar |  | EU191846 | EU191878 | JX263710 | JX264027 | JX263916 |
| PoPol14 | Polygraphini | Polygraphus coronatus | Cameroon | Limbe, Bonadikombe | JX263853 | JX264135 | JX263755 | JX264062 | JX263958 |
| PoPol04 | Polygraphini | Polygraphus poligraphus | Sweden | Åre | JX263851 | JX264134 | JX263753 | JX264060 | JX263956 |
| PoGen01 | Polygraphini | Polygraphus pseudobrunneus | Cameroon | Limbe, Bimbia | JX263849 | JX264132 | JX263751 | - | JX263955 |
| PoPol06 | Polygraphini | Polygraphus rufipennis | USA | NC, Blueridge Pkway | JX263852 | AF308407 | JX263754 | JX264061 | JX263957 |
| PoGen02 | Polygraphini | Polygraphus sp. E | Cameroon | Limbe, Bonadikombe | JX263850 | JX264133 | JX263752 | - | - |
| PoSer01 | Polygraphini | Serrastus similis | Cameroon | Limbe, Ekonjo | JX263854 | JX264136 | JX263756 | - | JX263959 |
| XyPre01 | Premnobiini | Premnobius cavipennis | South Africa | E. Cape Prov, Grahamstown | HQ883694 | HQ883762 | HQ883605 | HQ883839 | HQ883925 |
| PrPre01 | Premnobiini | Premnobius sp. | Sierra Leone | Tiwai Island | JX263855 | JX264137 | - | JX264063 | JX263960 |
| ScCam02 | Scolytini | Camptocerus aenipennis | Guyana | Iwokrama | HQ883676 | HQ883745 | HQ883587 | HQ883818 | HQ883907 |
| ScCam01 | Scolytini | Camptocerus auriconis | Costa Rica |  | EU191864 | EU191896 | HQ883586 | HQ883817 | HQ883906 |
| ScCne01 | Scolytini | Cnemonyx vismiaecolens | Guyana | Iwokrama | EU191865 | EU191897 | HQ883588 | HQ883819 | HQ883908 |
| ScScl02 | Scolytini | Scolytus intricatus | Sweden | Oskarshamn | HQ883677 | HQ883746 | HQ883589 | HQ883820 | HQ883909 |
| ScScl06 | Scolytini | Scolytus scolytus | Denmark | NEJ, Tofte Skov | HQ883678 | HQ883747 | HQ883590 | HQ883821 | HQ883910 |
| SpScp01 | Scolytoplatypodini | Scolytoplatypus africanus | Uganda | Kibale | EU191866 | EU191898 | - | HQ883822 | - |
| SpScp04 | Scolytoplatypodini | Scolytoplatypus entomoides | Papua New Guinea |  | HQ883679 | HQ883748 | EU090345 | HQ883823 | - |
| SpScp03 | Scolytoplatypodini | Scolytoplatypus tycon | Japan |  | JX263861 | JX264142 | JX263764 | - | - |
| XyXyl02 | Xyleborini | Anisandrus dispar | Norway | Oklungen, Porsgrunn | HQ883695 | HQ883763 | HQ883606 | HQ883840 | HQ883926 |
| XyCne00 | Xyleborini | Cnestus bimaculatus | Thailand |  | GU808695 | - | GU808579 | GU808619 | GU808657 |
| XyXyl00 | Xyleborini | Xyleborus affinis | Uganda | Kibale Forest, S. Fort Portal | AF187138 | AF186688 | GU808581 | GU808621 | GU808659 |
| XcCto03 | Xyloctonini | Ctonoxylon flavescens | Uganda | Masindi | AY376998 | AY377062 | JX263775 | JX264071 | JX263979 |
| XcXyc02 | Xyloctonini | Ctonoxylon methneri | South Africa | WC: Knysna, Diepwalle | JX263873 | - | - | - | - |
| CrSph03 | Xyloctonini | Glostatus sp H | South Africa | WC: Nature's Valley | EU191843 | EU191875 | JX263700 | - | - |
| XcCry01 | Xyloctonini | Glostatus sp. 1 | Cameroon | Limbe, Bonadikombe | JX263868 | JX264148 | JX263773 | - | JX263978 |
| XcCry02 | Xyloctonini | Glostatus sp. 2 - 'spiny' | Tanzania | Mang'ula: Megombera Forest | JX263869 | JX264149 | JX263774 | JX264070 | **-** |
| XcGlo04 | Xyloctonini | Glostatus sp. near xyloctonus | Tanzania | Udzungwa, Sanje trail, 700 m alt | JX263870 | - | JX263776 | - | JX263980 |
| CrSph01 | Xyloctonini | Glostatus squamosus | South Africa | WC: Wilderness | EU191842 | EU191874 | - | - | - |
| XcScm01 | Xyloctonini | Scolytomimus phillipinensis | Papua New Guinea | Madang, Beitata | JX263871 | JX264150 | JX263777 | - | JX263981 |
| XcXyc01 | Xyloctonini | Xyloctonus maculatus | South Africa | WC: Nature's Valley | JX263872 | JX264151 | JX263778 | - | JX263982 |
| XtInd01 | Xyloterini | Indocryphalus pubipennis | Japan |  | HQ883693 | AF375276 | HQ883604 | HQ883836 | - |
| XtTry02 | Xyloterini | Trypodendron domesticum | Norway | Trondheim | JX263874 | JX264152 | JX263779 | JX264072 | JX263983 |
| XtTry01 | Xyloterini | Trypodendron lineatum | Norway/ USA | Trondheim / | AF187132 | AF186682 | AF308394 | HQ883837 | HQ883923 |
| XtXyl01 | Xyloterini | Xyloterinus politus | USA | NH: Mt. Monadnock | AF187133 | AF186683 | AF308395 | HQ883838 | HQ883924 |
