# Supplementary material for: Molecular phylogeny of bark and ambrosia beetles reveals multiple origins of fungus farming during periods of global warming
Source: BMC Evol Biol. 2012 Aug 1;12:133. doi: 10.1186/1471-2148-12-133 (PMC3514184; doi:10.1186/1471-2148-12-133)
Supplement: Additional file 4 — Table S2. Character states for the taxa sampled, with the absence (0) or presence (1) of fungus farming, gregarious larval feeding and regular inbreeding. [file 1471-2148-12-133-S4.docx]

Table S2. Character states for taxa included in the phylogenetic analyses of Scolytinae, including the absence (0)or presence (1) of fungus farming, gregarious feeding and regular inbreeding by sibling mating.

| **Code** | **Tribe** | **Species** | **Fungus farming** | **Larvae gregarious** | **Sibling mating** |
| --- | --- | --- | --- | --- | --- |
| 0CsXxx01 | Conoderinae | Homoeometamlus spA | 0 | 0 | 0 |
| 0EnPol01 | Entiminae | Polydrusus cervinus | 0 | 0 | 0 |
| 0MoPor01 | Molytinae | Porthetes hispidus | 0 | 0 | 0 |
| BoBot01 | Bothrosternini | Bothrosternus foveatus | 1 | 1 | 1 |
| BoCne01 | Bothrosternini | Cesinus lecontei | 0 | 1 | 0 |
| BoEup01 | Bothrosternini | Eupagiocerus dentipes | 1 | 1 | 0 |
| BoSte01 | Bothrosternini | Sternobothrus sp. | 0 | 1 | 0 |
| CaCac01 | Cactopinini | Cactopinus rhois | 0 | 0 | 0 |
| CoAmp01 | Corthylini | Amphicranus sp. | 1 | 0 | 0 |
| CoAmp02 | Corthylini | Amphicranus sp. B | 1 | 0 | 0 |
| CoAra01 | Corthylini | Araptus declivis | 0 | 0 | 0 |
| CoCor01 | Corthylini | Corthylus rubricollis | 1 | 0 | 0 |
| CoDen01 | Corthylini | Dendroterus defectus | 0 | 0 | 0 |
| CoMim02 | Corthylini | Genus? | 0 | 0 | 0 |
| CoXxx01 | Corthylini | Genus? | ? | 0 | 0 |
| CoGna01 | Corthylini | Gnathotrichus materiarius | 1 | 0 | 0 |
| CoMon01 | Corthylini | Monarthrum mali | 1 | 0 | 0 |
| CoPit01 | Corthylini | Pityophtorus micrographus | 0 | 0 | 0 |
| CoPsp01 | Corthylini | Pseudopityophthorus yavapaii | 0 | 0 | 0 |
| CoTri01 | Corthylini | Tricolus sp.A | 1 | 0 | 0 |
| CoTri02 | Corthylini | Tricolus sp.B | 1 | 0 | 0 |
| CrAll01 | Cryphalini | Allernoporus euonymi | 0 | 0 | 0 |
| CrPti01 | Cryphalini | Cosmoderes sp. 1 | 0 | 0 | 0 |
| CrCos01 | Cryphalini | Cosmoderes sp. 2 | 0 | 0 | 0 |
| CrCry02 | Cryphalini | Cryphalus abietis | 0 | 1 | 0 |
| CrCry04 | Cryphalini | Cryphalus longulus | 0 | 1 | 0 |
| CrEcl01 | Cryphalini | Cryphalus? sp. C | 0 | 1 | 0 |
| CrErn04 | Cryphalini | Ernoporicus spessivtzevi | 0 | 0 | 0 |
| CrErn05 | Cryphalini | Ernoporus eggersi | 0 | 0 | 0 |
| CrErn03 | Cryphalini | Ernoporus tiliae | 0 | 0 | 0 |
| CrHyp02 | Cryphalini | Hypothenemus birmanus | 0 | 1 | 1 |
| CrHyp01 | Cryphalini | Hypothenemus nr. not eruditus | 1 | 1 | 1 |
| CrCry05 | Cryphalini | Procryphalus fraxini | 0 | 0 | 0 |
| CrPro01 | Cryphalini | Procryphalus mucronatus | 0 | 0 | 0 |
| CrPti02 | Cryphalini | Ptilopodius sp. | 0 | 1 | 1 |
| CrXxx02 | Cryphalini | Scolytogenes nr onyanganus | 0 | 0 | 0 |
| CrXxx01 | Cryphalini | Scolytogenes sp. | 0 | 0 | 0 |
| CrTry01 | Cryphalini | Trypophloeus alni | 0 | 0 | 0 |
| CrTry04 | Cryphalini | Trypophloeus tremulae | 0 | 0 | 0 |
| CgAph02 | Crypturgini | Aphanarthrum capense | 0 | 0 | 0 |
| CgCol01 | Crypturgini | Aphanarthrum maculatum | 0 | 0 | 0 |
| CgCis01 | Crypturgini | Cisurgus wollastoni | 0 | 0 | 0 |
| CgCol03 | Crypturgini | Coleobothrus luridus | 0 | 0 | 0 |
| CgCryAlu | Crypturgini | Crypturgus alutaceus | 0 | 0 | 0 |
| CgCry02 | Crypturgini | Crypturgus borealis | 0 | 0 | 0 |
| CgCry01 | Crypturgini | Crypturgus cinereus | 0 | 0 | 0 |
| CgDol01 | Crypturgini | Dolurgus pumilus | 0 | 0 | 0 |
| DiAca01 | Diamerini | Acacicis minor | 0 | 0 | 0 |
| DiDia01 | Diamerini | Diamerus curvifer | 0 | 0 | 0 |
| DiDia03 | Diamerini | Diamerus inermis | 0 | 0 | 0 |
| DiSph01 | Diamerini | Sphaerotrypes hagedorni | 0 | 0 | 0 |
| DiStr02 | Diamerini | Strombophorus capensis | 0 | 0 | 0 |
| DiStr03 | Diamerini | Strombophorus celtis | 0 | 0 | 0 |
| DiStr04 | Diamerini | Strombophorus sp. not elongatus | 0 | 0 | 0 |
| DrCoc01 | Dryocoetini | Coccotrypes dactyliperda | 0 | 1 | 1 |
| DrCoc00 | Dryocoetini | Coccotrypes longior | 0 | 1 | 1 |
| DrCyr01 | Dryocoetini | Cyrtogenius africus | 0 | 0 | 0 |
| DrDac01 | Dryocoetini | Dactylotrypes longicollis | 0 | 1 | 0 |
| DrDry02 | Dryocoetini | Dryocoetes alni | 0 | 0 | 0 |
| DrDry01 | Dryocoetini | Dryocoetes autographus | 0 | 0 | 0 |
| DrDrc02 | Dryocoetini | Dryocoetiops coffea | 0 | 1 | 1 |
| DrDrc01 | Dryocoetini | Dryocoetiops petioli | 0 | 0 | 0 |
| DrLym02 | Dryocoetini | Lymantor aceris | 0 | 0 | 0 |
| DrLym01 | Dryocoetini | Lymantor coryli | 0 | 0 | 0 |
| DrOzo02 | Dryocoetini | Ozopemon uniseriatus | 0 | 1 | 1 |
| DrTap02 | Dryocoetini | Taphrorychus bicolor | 0 | 0 | 0 |
| DrTap01 | Dryocoetini | Taphrorychus villifrons | 0 | 0 | 0 |
| DrCyr03 | Dryocoetini | Thamnurgus ?cylindricus | 0 | 0 | 0 |
| DrThm01 | Dryocoetini | Thamnurgus senicionis | 0 | 0 | 0 |
| DrTri01 | Dryocoetini | Triotemnus subretusus | 0 | 0 | 0 |
| DrXyl03 | Dryocoetini | Xylocleptes adeniae | 0 | 0 | 0 |
| DrXyl01 | Dryocoetini | Xylocleptes bispinus | 0 | 0 | 0 |
| CtGym01 | Hexacolini | Gymnochilus reitteri | 0 | 0 | 0 |
| CtMic01 | Hexacolini | Microborus cf. boops | 0 | 0 | 0 |
| CtMic03 | Hexacolini | Microborus sp. | 0 | 0 | 0 |
| CtPyc01 | Hexacolini | Pycnarthrum hispidum 1 | 0 | 0 | 0 |
| CtPyc012 | Hexacolini | Pycnarthrum hispidum 2 | 0 | 0 | 0 |
| CtSct01 | Hexacolini | Scolytodes acuminatus | 1 | 0 | 0 |
| CtSct04 | Hexacolini | Scolytodes sp. | 0 | 0 | 0 |
| HtHyt02 | Hylastini | Hylastes brunneus | 0 | 0 | 0 |
| HtHyt05 | Hylastini | Hylastes opacus | 0 | 0 | 0 |
| HtHyt08 | Hylastini | Hylastes porculus | 0 | 0 | 0 |
| HtHyg02 | Hylastini | Hylurgops glabratus | 0 | 0 | 0 |
| HtHyg09 | Hylastini | Hylurgops rugipennis | 0 | 0 | 0 |
| HlAln01 | Hylesinini | Alniphagus aspericollis | 0 | 0 | 0 |
| HlDac01 | Hylesinini | Dactylipalpus grouvellei | 0 | 0 | 0 |
| HlFic02 | Hylesinini | Ficicis despectus | 0 | 0 | 0 |
| HlFic01 | Hylesinini | Ficicis wallacei | 0 | 0 | 0 |
| HlHap02 | Hylesinini | Hapalogenius oblongus | 0 | 0 | 0 |
| HlHap01 | Hylesinini | Hapalogenius pusillus | 0 | 0 | 0 |
| HlHlt02 | Hylesinini | Hylastini fankhauseri | 0 | 0 | 0 |
| HlHnp03 | Hylesinini | Hylesinopsis dubius | 0 | 0 | 0 |
| HlHnp01 | Hylesinini | Hylesinopsis fasciatus | 0 | 0 | 0 |
| HlHnp02 | Hylesinini | Hylesinopsis granulatus | 0 | 0 | 0 |
| HlHyl01 | Hylesinini | Hylesinus toranio | 0 | 0 | 0 |
| HlHyl02 | Hylesinini | Hylesinus varius | 0 | 0 | 0 |
| HlPhb02 | Hylesinini | Phloeoborus sp. | 0 | 0 | 0 |
| HlPte02 | Hylesinini | Pteleobius vittatus | 0 | 0 | 0 |
| HlRho01 | Hylesinini | Rhopalopselion thompsoni | 0 | 0 | 0 |
| ToCha02 | Hylurgini | Chaetoptelius tricolor | 0 | 0 | 0 |
| ToCha01 | Hylurgini | Chaetoptelius vestitus | 0 | 0 | 0 |
| ToDen01 | Hylurgini | Dendroctonus micans | 0 | 1 | 1 |
| ToDen02 | Hylurgini | Dendroctonus terebrans | 0 | 0 | 0 |
| ToHdr01 | Hylurgini | Hylurdrectonus corticinus | 0 | 0 | 0 |
| ToHdr02 | Hylurgini | Hylurdrectonus pinarius | 0 | 0 | 0 |
| ToHgn01 | Hylurgini | Hylurgonotus antipodus | 0 | 0 | 0 |
| ToHgn02 | Hylurgini | Hylurgonotus tuberculatus | 0 | 0 | 0 |
| ToHrg01 | Hylurgini | Hylurgopinus rufipes | 0 | 0 | 0 |
| ToHyr01 | Hylurgini | Hylurgus ligniperda | 0 | 0 | 0 |
| ToHyr02 | Hylurgini | Hylurgus micklitzi | 0 | 0 | 0 |
| ToPac01 | Hylurgini | Pachycotes grandis | 0 | 0 | 0 |
| ToPsh01 | Hylurgini | Pseudohylesinus nebulosus | 0 | 0 | 0 |
| ToSin01 | Hylurgini | Sinophloeus destructor | 0 | 0 | 0 |
| ToTom01 | Hylurgini | Tomicus piniperda | 0 | 0 | 0 |
| ToXch01 | Hylurgini | Xylechinosomus valdivianus | 0 | 0 | 0 |
| ToXyl02 | Hylurgini | Xylechinus araucariae | 0 | 0 | 0 |
| PtPht02 | Hylurgini | Xylechinus maculatus | 0 | 0 | 0 |
| ToXyl01 | Hylurgini | Xylechinus pilosus | 0 | 0 | 0 |
| HcSue01 | Hyorrhynchini | Sueus niisimai | 1 | 1 | 1 |
| HyCha01 | Hypoborini | Chaetophloeus heterodoxus | 0 | 0 | 0 |
| HyCha02 | Hypoborini | Chaetophloeus penicillatus | 0 | 0 | 0 |
| CrCh?01 | Hypoborini | Genus? | 0 | 0 | 0 |
| HyHyb01 | Hypoborini | Hypoborus ficus | 0 | 0 | 0 |
| HyLip01 | Hypoborini | Liparthrum australis | 0 | 0 | 0 |
| HyLip02 | Hypoborini | Liparthrum nigrescens | 0 | 0 | 0 |
| HySty01 | Hypoborini | Styracoptinus euphorbiae | 0 | 0 | 0 |
| IpAca01 | Ipini | Acanthotomicus sp. 1 | 0 | 0 | 0 |
| DrCyr02 | Ipini | Acanthotomicus sp. 2 | 0 | 0 | 0 |
| DrCyr04 | Ipini | Acanthotomicus sp. 3 | 0 | 0 | 0 |
| IpAca02 | Ipini | Acanthotomicus tanganyikiensis | 0 | 0 | 0 |
| IpIps02 | Ipini | Ips acuminatus | 0 | 0 | 0 |
| IpIps03 | Ipini | Ips duplicatus | 0 | 0 | 0 |
| IpIps01 | Ipini | Ips typographus | 0 | 0 | 0 |
| IpOrt01 | Ipini | Orthotomicus proximus | 0 | 1 | 0 |
| IpPit01 | Ipini | Pityogenes bistridentatus | 0 | 0 | 0 |
| IpPit03 | Ipini | Pityogenes quadridens | 0 | 0 | 0 |
| IpPtk01 | Ipini | Pityokteinus minutus | 0 | 0 | 0 |
| IpPse01 | Ipini | Pseudips mexicanus | 0 | 1 | 0 |
| CrCh?04 | Micracidini | Genus? | 0 | 0 | 0 |
| CrEcl02 | Micracidini | Genus? | 0 | 0 | 0 |
| MiHyl01 | Micracidini | Hylocurus femineus | 0 | 0 | 0 |
| MiLan02 | Micracidini | Lanurgus sp. G | 0 | 0 | 0 |
| MiTra01 | Micracidini | Lanurgus sp. N | 0 | 0 | 0 |
| MiLan01 | Micracidini | Lanurgus xylographus | 0 | 0 | 0 |
| MiMic01 | Micracidini | Micracis carinulatus | 0 | 0 | 0 |
| MiMio02 | Micracidini | Miocryphalus congonus | 0 | 0 | 0 |
| MiMio01 | Micracidini | Miocryphalus sp. C | 0 | 0 | 0 |
| MiMio03 | Micracidini | Miocryphalus? sp. B | 0 | 0 | 0 |
| MiPse01 | Micracidini | Pseudothysanoes cf. leechi | 0 | 0 | 0 |
| PhChr01 | Phloeosinini | Chramesus asperatus | 0 | 0 | 0 |
| PhDen01 | Phloeosinini | Dendrosinus globosus | 0 | 0 | 0 |
| PhHyl02 | Phloeosinini | Hyledius nitidicollis | 0 | 0 | 0 |
| PhHyl01 | Phloeosinini | Hyledius vilis | 0 | 0 | 0 |
| PtNew01 | Phloeosinini | Microditica uniseriata | 0 | 0 | 0 |
| PhPhd01 | Phloeosinini | Phloeoditica curta | 0 | 0 | 0 |
| PhPps01 | Phloeosinini | Phloeosinopsioides formosanus | 0 | 0 | 0 |
| PhPhl01 | Phloeosinini | Phloeosinus aubei | 0 | 0 | 0 |
| PhPhl03 | Phloeosinini | Phloeosinus punctatus | 0 | 0 | 0 |
| PhPch01 | Phloeosinini | Pseudochramesus acuteclavatus | 0 | 0 | 0 |
| PtPht05 | Phloeotribini | Phloeotribus scarabaeoides | 0 | 0 | 0 |
| PtPht01 | Phloeotribini | Phloeotribus spinulosus | 0 | 0 | 0 |
| PxPrx01 | Phrixosomini | Phrixosoma minor | 0 | 0 | 0 |
| PxPrx03 | Phrixosomini | Phrixosoma sp. ?minor | 0 | 0 | 0 |
| PxGen01 | Phrixosomini | Phrixosoma sp. nov. | 0 | 0 | 0 |
| PxPrx02 | Phrixosomini | Phrixosoma uniseriatum | 0 | 0 | 0 |
| PoCar03 | Polygraphini | Carphoborus bifurcus | 0 | 0 | 0 |
| PoCar01 | Polygraphini | Carphoborus perrisi | 0 | 0 | 0 |
| PoCar02 | Polygraphini | Carphoborus sp. | 0 | 0 | 0 |
| PoCho01 | Polygraphini | Chortastus medius | 0 | 0 | 0 |
| DrDol02 | Polygraphini | Dolurgocleptes malgassicus | 0 | 0 | 0 |
| DrDol01 | Polygraphini | Dolurgocleptes punctifer | 0 | 0 | 0 |
| PoPol14 | Polygraphini | Polygraphus coronatus | 0 | 0 | 0 |
| PoPol04 | Polygraphini | Polygraphus poligraphus | 0 | 0 | 0 |
| PoGen01 | Polygraphini | Polygraphus pseudobrunneus | 0 | 0 | 0 |
| PoPol06 | Polygraphini | Polygraphus rufipennis | 0 | 0 | 0 |
| PoGen02 | Polygraphini | Polygraphus sp. E | 0 | 0 | 0 |
| PoSer01 | Polygraphini | Serrastus similis | 0 | 0 | 0 |
| XyPre01 | Premnobiini | Premnobius cavipennis | 1 | 0 | 1 |
| PrPre01 | Premnobiini | Premnobius sp. | 1 | 0 | 1 |
| ScCam02 | Scolytini | Camptocerus aenipennis | 1 | 0 | 0 |
| ScCam01 | Scolytini | Camptocerus auriconis | 1 | 0 | 0 |
| ScCne01 | Scolytini | Cnemonyx vismiaecolens | 0 | 0 | 0 |
| ScScl02 | Scolytini | Scolytus intricatus | 0 | 0 | 0 |
| ScScl06 | Scolytini | Scolytus scolytus | 0 | 0 | 0 |
| SpScp01 | Scolytoplatypodini | Scolytoplatypus africanus | 1 | 0 | 0 |
| SpScp04 | Scolytoplatypodini | Scolytoplatypus entomoides | 1 | 0 | 0 |
| SpScp03 | Scolytoplatypodini | Scolytoplatypus tycon | 1 | 0 | 0 |
| XyXyl02 | Xyleborini | Anisandrus dispar | 1 | 1 | 1 |
| XyCne00 | Xyleborini | Cnestus bimaculatus | 1 | 1 | 1 |
| XyXyl00 | Xyleborini | Xyleborus affinis | 1 | 1 | 1 |
| XcCto03 | Xyloctonini | Ctonoxylon flavescens | 0 | 0 | 0 |
| XcXyc02 | Xyloctonini | Ctonoxylon methneri | 0 | 0 | 0 |
| CrSph03 | Xyloctonini | Glostatus sp H | 0 | 0 | 0 |
| XcCry01 | Xyloctonini | Glostatus sp. 1 | 0 | 0 | 0 |
| XcCry02 | Xyloctonini | Glostatus sp. 2 - 'spiny' | 0 | 0 | 0 |
| XcGlo04 | Xyloctonini | Glostatus sp. near xyloctonus | 0 | 0 | 0 |
| CrSph01 | Xyloctonini | Glostatus squamosus | 0 | 0 | 0 |
| XcScm01 | Xyloctonini | Scolytomimus phillipinensis | 0 | 0 | 0 |
| XcXyc01 | Xyloctonini | Xyloctonus maculatus | 0 | 0 | 0 |
| XtInd01 | Xyloterini | Indocryphalus pubipennis | 1 | 0 | 0 |
| XtTry02 | Xyloterini | Trypodendron domesticum | 1 | 0 | 0 |
| XtTry01 | Xyloterini | Trypodendron lineatum | 1 | 0 | 0 |
| XtXyl01 | Xyloterini | Xyloterinus politus | 1 | 0 | 1 |
