# Supplementary material for: Molecular phylogeny of bark and ambrosia beetles reveals multiple origins of fungus farming during periods of global warming
Source: BMC Evol Biol. 2012 Aug 1;12:133. doi: 10.1186/1471-2148-12-133 (PMC3514184; doi:10.1186/1471-2148-12-133)
Supplement: Additional file 5 — Table S3. Properties of each partition, estimated from 84 taxa with no missing data. [file 1471-2148-12-133-S5.docx]

Supplementary Table S3. Properties of each partition, estimated from a fully resolved tree topology resulting from the parsimony analysis of 84 taxa (no missing data).

| Partition | # taxa | Char. | PI | Steps* | CI* | Best model |
| --- | --- | --- | --- | --- | --- | --- |
|  |  |  |  |  |  |  |
| COI | 190 | 690 | 343 | 6872 | 0.10 | GTR+I+G |
| Coi1st |  | 230 | 29 | 1227 | 0.13 | GTR+I+G |
| Coi2nd |  | 230 | 33 | 271 | 0.24 | GTR+I+G |
| Coi3rd |  | 230 | 224 | 5376 | 0.09 | GTR+G |
| EF1a | 164 | 857 | 326 | 4882 | 0.13 | GTR+I+G |
| Ef1st |  | 286 | 44 | 437 | 0.17 | GTR+I+G |
| Ef2nd |  | 286 | 19 | 204 | 0.17 | GTR+I+G |
| Ef3rd |  | 285 | 263 | 4242 | 0.13 | GTR+I+G |
| CAD |  | 675 | 323 | 5776 | 0.12 | GTR+I+G |
| Cad1st |  | 225 | 71 | 690 | 0.17 | GTR+I+G |
| Cad2nd |  | 225 | 31 | 230 | 0.25 | GTR+I+G |
| Cad3rd |  | 225 | 221 | 4856 | 0.11 | GTR+I+G |
| ArgK |  | 801 | 345 | 4975 | 0.13 | GTR+I+G |
| Argk1st |  | 267 | 62 | 511 | 0.15 | GTR+I+G |
| Argk2nd |  | 267 | 24 | 193 | 0.19 | GTR+I+G |
| Argk3rd |  | 267 | 259 | 4271 | 0.12 | GTR+I+G |
| 28S |  | 671 | 265 | 2594 | 0.19 | GTR+I+G |

^*^based on informative characters only
